# Supplementary material for: Thermosipho spp. Immune System Differences Affect Variation in Genome Size and Geographical Distributions
Source: Genome Biol Evol. 2018 Sep 15;10(11):2853–66. doi: 10.1093/gbe/evy202 (PMC6211235; doi:10.1093/gbe/evy202)
Supplement: Supplementary Data [file evy202_supp.zip › Supplementary_Material_Figures.docx]

**Supplementary Material Figures S1 to S4.**

Figures are in order as mentioned in the main text.

**Supplementary Material Figure S1**. Maximum Likelihood phylogeny of full-length *Thermosipho* sp 16S rRNA gene sequences. The phylogeny is rooted using 16S rRNA gene sequences of representative sequences from other Thermotogales genera. The percentage of 1000 bootstrap replicates is shown for each branching node. Sequences with Bold fonts are whole genome sequences. Triangles behind the sequence ID indicate genome not from this study. Circles behind sequence ID indicate genome from this study.

**Supplementary Material Figure S2.** Recombination detection between 10 *Thermosipho* isolates using Likewind. Likewind uses a sliding window (1000bp) to perform a comparison of the Maximum log-likelihood of the best tree from the window data and the ML tree of the complete on concatenated LCB blocks (Mauve) alignment. The phylogenetic differences from this comparison are expressed as ΔLog L. **A**) Phylogenetic tree based on the whole alignment. **B, C & D**) Phylogenetic trees based on regions showing within species recombinations. **E**) ΔLog L dynamics for the concatenated LCB alignment. The x-axis presents the concatenated LCB alignment positions. The horizontal grey line indicates the cut-off value for phylogenies that are significantly different after parametric bootstrap analysis.


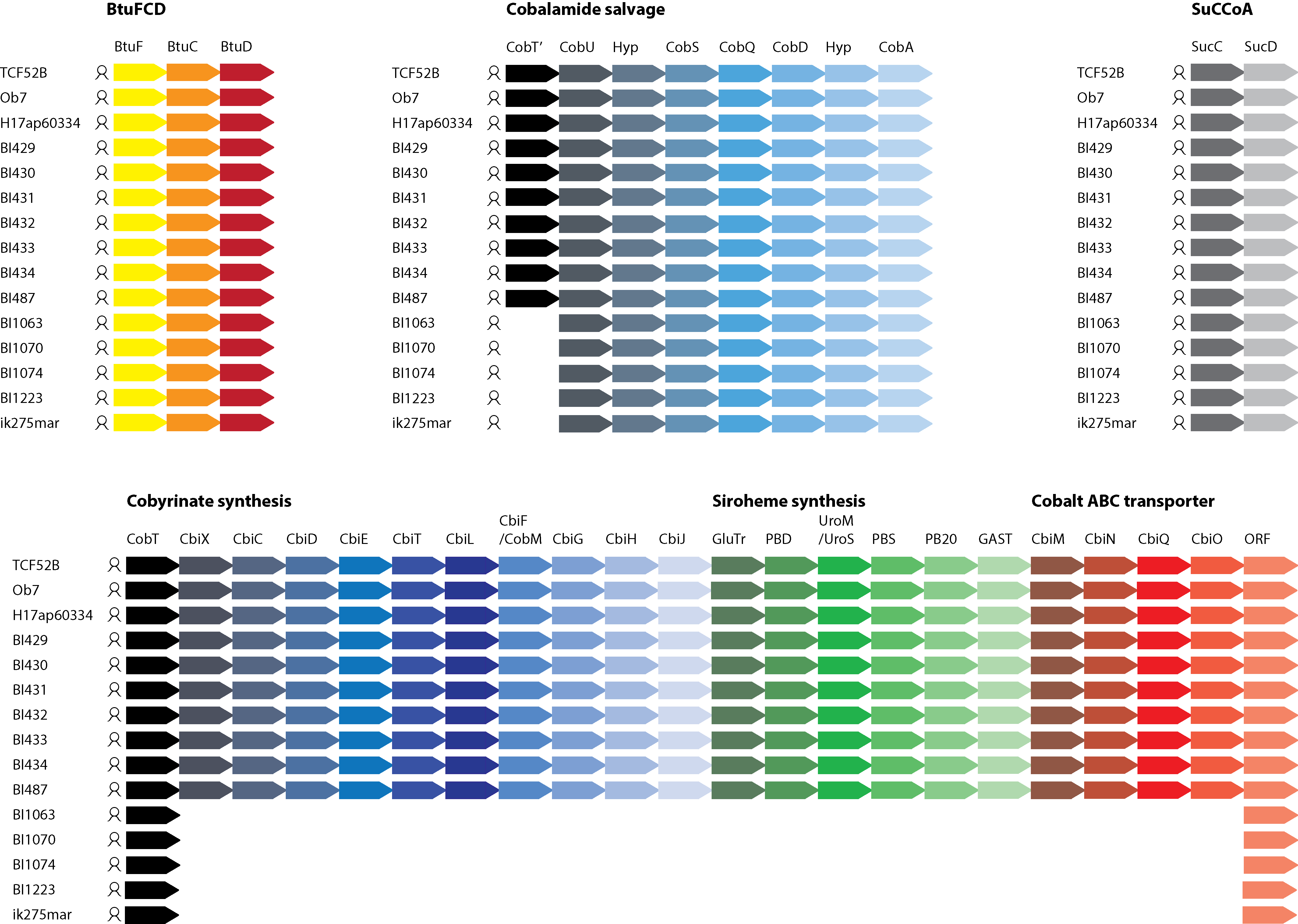


**Supplementary Material Figure S3.** Schematic representation of vitamine B_12_ biosynthesis pathway genes present in 15 *Thermosipho* sp. genomes. The vitamine B_12_ pathway genes are divided over four gene clusters in *Thermosipho*: BtuFCD, Corriniod, Cobalamin, and SucCoA and are regulated by riboswitches (Swithers et al. 2011). The corrinoid gene cluster consists of the cobyrinate and siroheme synthesis and Cobalt ABC transporter genes. Riboswitches are indicated with the inverted “♉” symbol. All 15 genomes were screened for the presence of Cobalamin specific riboswitches using Riboswitch scanner (Mukherjee & Sengupta 2015). Protein sequences from the *T. melanesiensis* BI429 genome involved in B_12_ metabolism were extracted (Swithers et al. 2011) and used to identify homologous genes in all *Thermosipho* genomes using tBLASTn with a maximum e-value 1.0^-20^. If a gene was present it is indicated as a box with an arrow.


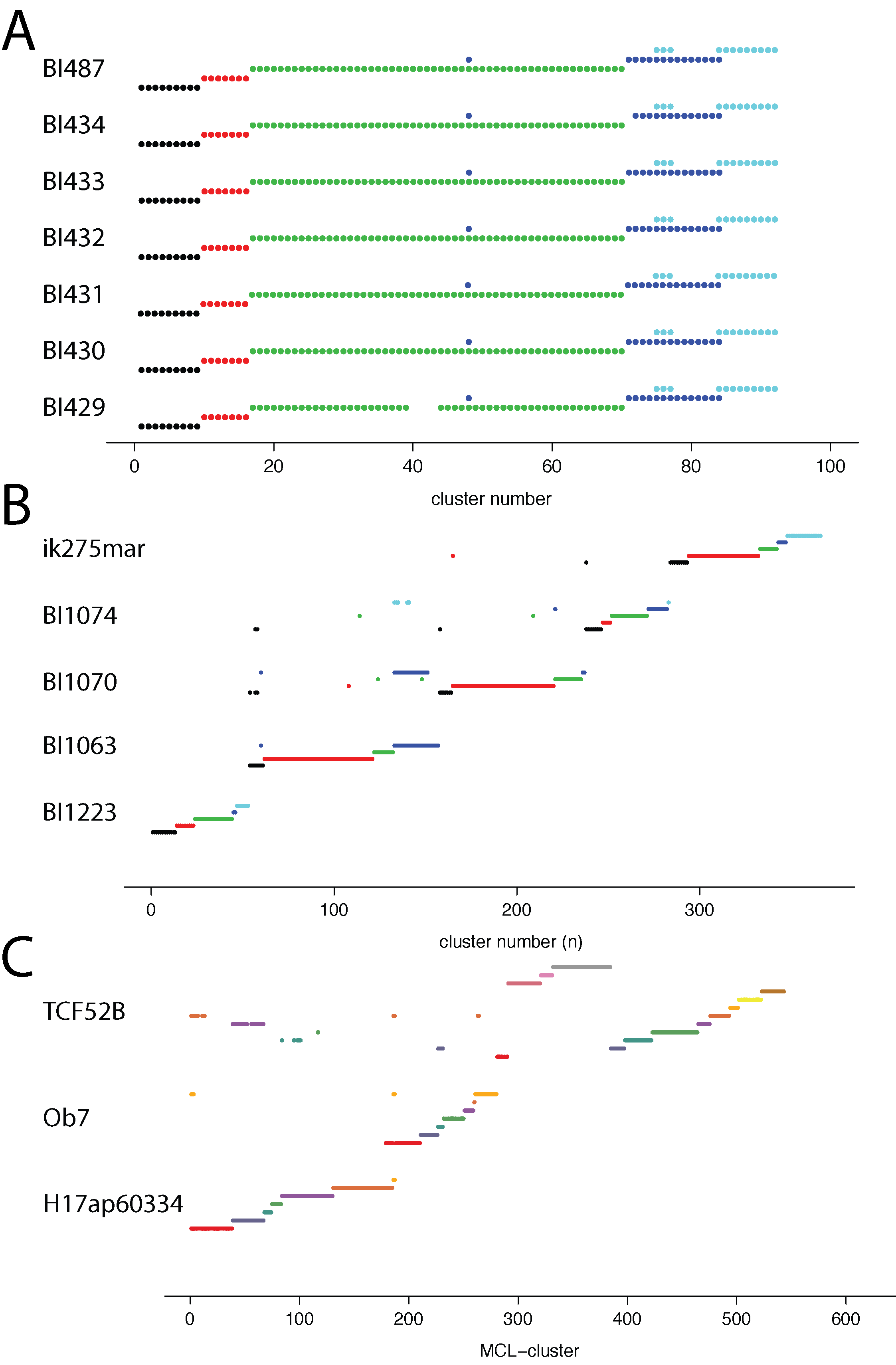


**Supplementary Material Figure S4.** CRISPR spacer comparison in three *Thermosipho* species. CRISPR spacers sequences in all genomes were extracted using CRISPR finder and compared using BLASTn. For each of the three species: *T. melanesiensis*, *T. affectus* and *T. africanus,* we clustered the CRISPR spacer sequences using MCL and igraph. MCL was run using the igraph distance matrix with the inflation set to 1.4 and max iterations set to 100 (Enright et al. 2002). This allowed for the identification of identical spacers within and between genomes. CRISPR spacers in the same CRISPR array are indicated with the same color. The x-axis represents the cluster ID of CRISPR spacers identified by MCL. **A)** Clustering of CRISPR spacers of *T. melanesiensis* isolates. **B)** Clustering of CRISPR spacers of *T. affectus* isolates. **C)** Clustering of CRISPR spacers of *T. africanus* isolates. There were no identical spacers identified between strains of different species.

**References**

Enright AJ, Van Dongen S, Ouzounis CA. 2002. An efficient algorithm for large-scale detection of protein families. Nucleic Acids Res. 30:1575–1584. doi: 10.1093/nar/30.7.1575.

Mukherjee S, Sengupta S. 2015. Riboswitch Scanner: an efficient pHMM-based web-server to detect riboswitches in genomic sequences. Bioinformatics. 32:776–778. doi: 10.1093/bioinformatics/btv640.

Swithers KS et al. 2011. Vitamin B(12) synthesis and salvage pathways were acquired by horizontal gene transfer to the *Thermotogales*. Genome Biology and Evolution. 4:730–739. doi: 10.1093/gbe/evs057.
