# Supplementary material for: Thermosipho spp. Immune System Differences Affect Variation in Genome Size and Geographical Distributions
Source: Genome Biol Evol. 2018 Sep 15;10(11):2853–66. doi: 10.1093/gbe/evy202 (PMC6211235; doi:10.1093/gbe/evy202)
Supplement: Supplementary Data [file evy202_supp.zip › Thermosipho_genomes_Supplementary_information_20180523.docx]

**Supplementary Information for: “*Thermosipho* spp. immune system differences affect variation in genome size and geographical distributions”**

***Material and Methods.***

*Blast Ring Image creation*

Blast Ring genome plots were generated using BRIG version 0.95 (Alikhan et al. 2011) running Blast+ version 2.2.28 (Camacho et al. 2009) using the following procedure for each genome. 1) The nucleotide sequence of one of the 15 *Thermosipho* genomes was used to create a reference genomes blast-database in BRIG with default settings. 2) Nucleotide sequences of all coding genes, predicted by the NCBI Genbank annotation, were extracted from each genome using CLC Main workbench Version 6.8.3 and were used for BLASTn analysis against the reference genome in BRIG with the following settings: max_target_seqs: 1; max e-value cut-off: 1.0^-4^. Alignments with a minimum of 70% similarity were visualized with BRIG. The same procedure was used for all genomes. One representative plot for each species is presented in the Supplementary materials Figure S3.

*Carbohydrate metabolism*

For each genome we extracted all the PFAM annotated genes. All PFAM annotations were compared against a local database of the dbCAN database (Yin et al. 2012) to extract the carbohydrate enzymes present in each genome. The results were tabulated and are represented in Supplementary materials Table S4.

*Vitamine B_12_ pathway analysis*

The genes involved in the Vitamine B_12_ metabolism are found in four different gene clusters (BtuFCD, Corriniod, Cobalamin, and SucCoA) in *Thermosipho* and can be regulated by B_12_ riboswitches (Swithers et al. 2011). All 15 genomes were screened for the presence of Cobalamin specific riboswitches using Riboswitch scanner (Mukherjee & Sengupta 2015). This information was used to confirm the presence of the four gene clusters in each genome. Next, we extracted the protein sequences from the *T. melanesiensis* BI429 genome involved in B_12_ metabolism (Swithers et al. 2011) and used them to identify homologous genes in all *Thermosipho* genomes using tBLASTn with a maximum e-value 1.0^-20^.

*CRISPR spacer clustering analysis*

CRISPR spacers sequences in all genomes were extracted using CRISPR finder and compared using BLASTn. For each of the three species: *T. melanesiensis*, *T. affectus* and *T. africanus,* we clustered the CRISPR spacer sequences using MCL and igraph (Enright et al. 2002). MCL was run using the igraph distance matrix with the inflation set to 1.4 and max iterations set to 100. This allowed for the identification of identical spacers within and between genomes.

*Prophage detection*

Prophages were identified in genomes using a combination of tools: Profinder, PHAST and PhySpi (Lima-Mendez et al. 2008; Zhou et al. 2011; Akhter et al. 2012). All three tools need a closed chromosome. Therefor, we used the union command in EMBOSS v. 6.5.7 on the unfinished genome to create pseudo-chromosomes (Rice et al. 2000). The “complete” genomes were analyzed with the three above tools, and the output was manually inspected. Putative prophage regions were checked for the presence of phage related annotations and manually corrected (Haverkamp et al, manuscript in prep).

**Supplementary materials – Results**

*BRIG vizualizations.*

The *T. melanesiensis* isolates are highly similar to each other, while the *T. africanus* and *T. affectus* show reduced similarity or absence of genes from their genomes compare to strain BI429. Interestingly, the region around 1 Mbp of the *T. melanesiensis BI429* genome shows a region (position 0.98 Mbp- 1.009 Mbp) of high similarity nucleotide sequences in the genomes of the two other species. This region contains 44 genes (2 tRNAs, and 42 coding genes), of which 27 genes are highly conserved housekeeping proteins involved in the assembly of the 30S and 50S ribosomal genes. All genes are in the same orientation (5’ to 3’), suggesting they are part of one operon. This particular region is shared by all three species and can be found in all 15 genomes (Supplementary materials – figures 1- 15).

*Crispr spacer analysis*

Between four and six CRISPR arrays were detected in all the genomes, except for *T. africanus* TCF52B, which contains 12 arrays (Table 2). The total number of spacers detected in all genomes was 1709 and were collapsed into 1001 different clusters (Supplementary material Figure S4). The highly similar *T. melanesiensis* genomes have 5 almost identical CRISPR arrays (n=681) with all spacers falling into 92 different clusters. The *T. affectus* spp. CRISPR array spacers (n=412) fall into 366 clusters, while the *T. africanus* spp. CRISPR array spacers (n= 616) form 543 clusters.

Comparing spacer sequences to the host genome (excluding CRISPR arrays, blastN e-value cut-off: 1.0^-5^) revealed a small fraction of the spacers with matches within its host genome (percentage identity 88-100%) (Table 5). The *T. affectus* genome spacers did not match with any region in the host genome. For the *T. melanesiensis* genomes one spacer (array 2, spacer 5) matched one gene (e.g. *T. melanesiensis* BI429: Tmel_1466: hypothetical protein) in all genomes (81.8% identity, 6 SNPs). This gene is located within a prophage element consisting of 50 protein coding genes (Tmel_1439 : Tmel_1486) (Haverkamp et al., in prep). The relatively low similarity between the spacer and the gene sequence could suggests that this gene is not or no longer a target sequence, but we can not exclude it (Cady & O'Toole 2011).

For the *T.* *africanus* genomes we detected one CRISPR spacer, shared by all three genomes, which has a non-perfect match to the same genomic region. The spacer matches a phospholipase / carboxylesterase gene (THA_1282 in strain TCF52B) with 89-92% identity. For *T.* *africanus* H17ap60334 we find three additional spacers with perfect matches in two of its gene; two spacers target H17ap60334_04822 and one targets H17ap60334_04912. Closer examination revealed that also these genes are part of a predicted prophage region (Haverkamp et al., in prep.).

Finally, we searched NCBI’s non-redundant nucleotide database for matches to the *Thermosipho* spacer sequences. This identified two identical 44 bp spacers, one in *T.* *africanus* H17ap60334 (array 2, spacer 7) and one in *T.* *africanus* TCF52B (array 5, spacer 20) that matched a sequence in the genome of *Pseudothermotoga* *elfii* DSM 9442 (genbank refseq ID: NC_022792). The identified region in that genome (bp 200095 – 200125) is a CRISPR spacer sequence of 38bp, which is identical to the *Thermosipho* spacer for 31 bases. Interestingly, the first six bases of the *Pseudothermotoga* spacer has no match to the *Thermosipho* H17ap60334 and TCF52B spacer, while the last 12 bases of the *Thermosipho* spacers have no match to the *Pseudothermotoga* spacer. This suggests the spacers in both species were acquired independently, but that they match a similar sequence.

*Genome-wide comparisons of COG categories*

In order to detect functional differences between the three species we compared their genomic content using Clusters of Orthologous Genes (COGs) annotations (Figure 4A). This revealed *T. africanus* to have genomes with the highest absolute gene abundances for many COG categories, which is due to the *T. africanus* genomes being larger. However, this effect disappears for most categories when using relative abundances of all COGs (Figure 4A; Supplementary Materials Table S3). The categories H, J, L, R and G had the largest relative abundance differences between the three species (p ≤ 0.01 for H, J and R and p ≤ 0.05 for G and L). Several other categories (B, C, I, O) had highly significant relative abundances differences (p ≤ 0.01), but absolute values were either very low (B), and relative abundance differences were not very large between the species, and within species they were very similar (Supplementary Materials Table S3).

The largest COG category is made up of Category R (General function prediction only), with the *T. affectus* genomes having most genes (Figure 4A). Category R is also the largest group among the species-specific genes (Supplementary Materials Table S3). These results are in line with the observation that on average 42.1 %, 49.3 % and 54.8 % (*T. africanus*, *T. affectus* and *T. melanesiensis*) of the species-specific genes lack COG annotations and are hypothetical genes. Interestingly, the *T. africanus* genomes have more genes with COG categories annotations, but they do show more genes with the COG category R. This difference could be caused by the careful manual curation of the TCF52B genome (Nesbø et al. 2009).

The *T. affectus* genomes show a significantly (p ≤ 0.01) lower relative abundance of genes in category H (Coenzyme transport and metabolism category) (Figure 4A). Closer inspection shows that the *T. affectus* genomes are lacking most of the genes (20 out of 22 genes) needed for corrinoid synthesis, except *CobT* and an ATP-binding protein (indicated as ORF). A complete set of corrinoid synthesis genes are found in the genomes of *T. africanus* and *T. melanesiensis*, and are essential for *de novo* vitamine B_12_ synthesis (Swithers et al. 2011) (Supplementary materials Figure S3). Interestingly, the cobalamide salvage pathway gene cluster, which is needed for retrieving incomplete corrinoid molecules from the environment, is present in the *T. affectus* genomes. This gene cluster is, however, missing its *CobT’* gene. This suggests that the orphan *CobT* gene, presumably a remnant from the missing corrinoid cluster, is now functioning in the cobalamide salvage pathway (Supplementary materials Figure S3).

Large differences among the genomes were also seen for COG category G (Carbohydrate transport and metabolism). In agreement with this, phenotypic differences in carbohydrate metabolism is one of the main other features, to distinguish between the three species (Podosokorskaya et al. 2011). Also for this category, we find that the *T. africanus* genomes have relatively more genes than the other two species (Figure 4A). This difference is even more pronounced for species-specific genes (Supplementary Materials Table S3B), where *T. africanus* genomes have more genes present in this category (Supplementary Materials Table S3B). A screening of the genomes using PFAM annotations and the carbohydrate database dbCAN (Yin et al. 2012), showed a similar pattern as with the COG annotations (Supplementary materials Table S3). The *T. affectus, T. melanesiensis and T. africanus* genomes contain on average: 16-17, 20 and 21-26 genes respectively that are involved in breakdown of carbohydrates (Supplementary materials Table S3). Moreover, the families, containing enzymes involved in breakdown of various beta-linked oligo- and polysaccharides (eg. cellulose, xylan, laminarin, lichenan, mannans and chitin) were found exclusively among the representatives of *T. africanus*. This shows, in line with the COG analysis, that the *T. africanus* species might be more versatile with regard to carbohydrate uptake and metabolism.

When we compared the COG categories for the species-specific genes we found even larger differences for many categories (Figure 4A). Since the *T. africanus* genomes have almost twice as many species-specific genes compared to the other two species, they also have proportionally more species-specific genes in most categories. For instance, for COG category L (Replication, recombination and repair) we find large variation in the number of genes among the three *T. africanus* strains, with TCF52B having relatively more of these genes compared to the other genomes (8.7 % vs 5.7%). Examination of the genes assigned to this category revealed that this difference is mainly due to the presence of 18 copies of transposases in the TCF52B genome.

**References**

Akhter S, Aziz RK, Edwards RA. 2012. PhiSpy: a novel algorithm for finding prophages in bacterial genomes that combines similarity- and composition-based strategies. Nucleic Acids Res. 40:e126. doi: 10.1093/nar/gks406.

Alikhan N-F, Petty NK, Ben Zakour NL, Beatson SA. 2011. BLAST Ring Image Generator (BRIG): simple prokaryote genome comparisons. BMC Genomics. 12:402. doi: 10.1186/1471-2164-12-402.

Cady KC, O'Toole GA. 2011. Non-identity-mediated CRISPR-bacteriophage interaction mediated via the Csy and Cas3 proteins. J Bacteriol. 193:3433–3445. doi: 10.1128/JB.01411-10.

Camacho C et al. 2009. BLAST+: architecture and applications. BMC Bioinformatics. 10:421. doi: 10.1186/1471-2105-10-421.

Enright AJ, Van Dongen S, Ouzounis CA. 2002. An efficient algorithm for large-scale detection of protein families. Nucleic Acids Res. 30:1575–1584. doi: 10.1093/nar/30.7.1575.

Lima-Mendez G, van Helden J, Toussaint A, Leplae R. 2008. Prophinder: a computational tool for prophage prediction in prokaryotic genomes. Bioinformatics. 24:863–865. doi: 10.1093/bioinformatics/btn043.

Mukherjee S, Sengupta S. 2015. Riboswitch Scanner: an efficient pHMM-based web-server to detect riboswitches in genomic sequences. Bioinformatics. 32:776–778. doi: 10.1093/bioinformatics/btv640.

Nesbø CL et al. 2009. The genome of *Thermosipho africanus* TCF52B: lateral genetic connections to the *Firmicutes* and *Archaea*. J Bacteriol. 191:1974–1978. doi: 10.1128/JB.01448-08.

Podosokorskaya OA, Kublanov IV, Reysenbach A-L, Kolganova TV, Bonch-Osmolovskaya EA. 2011. *Thermosipho affectus* sp. nov., a thermophilic, anaerobic, cellulolytic bacterium isolated from a Mid-Atlantic Ridge hydrothermal vent. Int J Syst Evol Microbiol. 61:1160–1164. doi: 10.1099/ijs.0.025197-0.

Rice P, Longden I, Bleasby A. 2000. EMBOSS: the European Molecular Biology Open Software Suite. Trends Genet. 16:276–277.

Swithers KS et al. 2011. Vitamin B(12) synthesis and salvage pathways were acquired by horizontal gene transfer to the *Thermotogales*. Genome Biology and Evolution. 4:730–739. doi: 10.1093/gbe/evs057.

Yin Y et al. 2012. dbCAN: a web resource for automated carbohydrate-active enzyme annotation. Nucleic Acids Res. 40:W445–W451. doi: 10.1093/nar/gks479.

Zhou Y, Liang Y, Lynch KH, Dennis JJ, Wishart DS. 2011. PHAST: a fast phage search tool. Nucleic Acids Res. 39:W347–52. doi: 10.1093/nar/gkr485.
